# Integration of Axillary Meristem Development and Gibberellin Homeostasis by *CjLAS* Regulates Shoot Branching in *Clerodendrum japonicum*

**DOI:** 10.3390/plants15182827

**Published:** 2026-09-15

**Authors:** Huajie Chen, Yanwen Deng, Congcong Wang, Guihua Liao, Yongbin Long, Lingye Su, Chunmei He, Mingfeng Xu, Hongfeng Wang

**Affiliations:** Guangdong Academy of Forestry/Guangdong Provincial Key Laboratory of Forest Silviculture, Protection and Utilizaton/Key Laboratory of National Forestry and Grassland Administration on Ecosystem Conservation and Restoration in the Guangdong-Hong Kong-Macao Cireater Bay Area, Guangzhou 510520, China

**Keywords:** *Clerodendrum japonicum*, branching regulation, gibberellin pathway, *CjLAS*, hormone-induced development

## Abstract

Branching architecture is a key agronomic and ornamental trait in woody plants; however, the molecular mechanisms coordinating hormone-mediated axillary bud formation and outgrowth remain largely unclear. *Clerodendrum japonicum* (Thunb.) Sweet is an important ornamental woody species with strong branching potential, but the regulatory network underlying its shoot branching has not been characterized. In this study, we investigated the regulatory basis of hormone-induced branching in *Clerodendrum japonicum* and identified a LATERAL SUPPRESSOR homolog, *CjLAS*, as a key candidate regulator. Combined application of gibberellic acid (GA3) and 6-benzyladenine (6-BA) significantly promoted axillary bud formation and outgrowth, accompanied by extensive transcriptional reprogramming. Transcriptome sequencing and weighted gene co-expression network analysis (WGCNA) revealed that *CjLAS* was closely associated with branching development and hormone-responsive pathways. Expression analysis showed that *CjLAS* expression increased during the progression of hormone-induced axillary bud development and branch formation. Ectopic overexpression of *CjLAS* in *Arabidopsis thaliana* enhanced shoot branching, confirming its conserved positive role in regulating plant architecture. Furthermore, overexpression of *CjLAS* repressed the gibberellin (GA) biosynthetic genes *AtGA20ox2* and *AtGA3ox2*, induced the catabolic gene *AtGA2ox6*, and reduced the endogenous levels of bioactive GA1 and GA4. These findings suggest that *CjLAS* promotes shoot branching by integrating axillary meristem regulation with GA homeostasis. This study reveals an additional layer of LAS-mediated regulation linking meristem development and hormone homeostasis during shoot branching in woody plants and provides insights into the molecular regulation of plant architecture in *C. japonicum*.

## 1. Introduction

*Clerodendrum japonicum* (Thunb.) Sweet, a perennial shrub of the Lamiaceae family, is widely distributed throughout southern China and Southeast Asia [1]. The species has both medicinal and ornamental value because of its abundant bioactive metabolites, prolonged flowering period, and conspicuous inflorescences [2,3]. As a cultivated ornamental and medicinal plant, its plant architecture strongly influences canopy development, biomass accumulation, and ornamental quality. Among architectural traits, shoot branching is particularly important because it determines the number and spatial distribution of lateral shoots [4]. Therefore, understanding the developmental and molecular mechanisms controlling shoot branching is important for elucidating plant architecture and improving the cultivation of *C. japonicum*.

Shoot branching is a fundamental architectural trait that profoundly influences plant productivity, biomass accumulation, and commercial value across diverse plant species. In cereal crops, branching-related traits such as tillering determine panicle number and ultimately grain yield [5,6]. In medicinal and ornamental plants, branch abundance directly affects canopy structure, biomass production, and aesthetic quality [7,8,9,10]. Developmentally, branching originates from axillary meristems and proceeds through two tightly coordinated phases: axillary meristem initiation and subsequent bud outgrowth [11,12,13]. Consequently, deciphering the regulatory mechanisms controlling axillary meristem establishment and bud formation has become a central objective in plant developmental biology and crop architecture research [14,15].

Shoot branching is governed by a sophisticated hormonal regulatory network that integrates endogenous developmental cues with environmental signals. Among the major phytohormones, auxin suppresses axillary bud outgrowth through the maintenance of apical dominance [16,17,18], whereas cytokinins (CKs) promote bud formation and stimulate branch formation [19,20]. In contrast, strigolactones function predominantly as negative regulators of branching by triggering the MAX2–SMXL signaling cascade [21,22,23]. Compared with these well-characterized hormones, the role of gibberellins (GAs) in branching regulation remains considerably more complex and context dependent. Classical studies have generally associated GAs with stem elongation and branch suppression. However, accumulating evidence indicates that GAs may either inhibit or promote branching depending on species, developmental stage, and tissue context, partly through interactions with DELLA proteins and the branching regulator *BRANCHED1* (*BRC1*) [24,25]. These findings suggest that branching outcomes are determined by dynamic crosstalk among GA, cytokinin, auxin, and strigolactone signaling pathways rather than by the action of a single hormone [26,27,28]. Nevertheless, the molecular mechanisms through which GA signaling influences branching in woody ornamental species remain poorly understood [29].

To translate hormonal signals into developmental outputs, plants rely on a hierarchy of transcriptional regulators that orchestrate axillary meristem initiation and bud growth. Among these regulators, *LATERAL SUPPRESSOR* (*LAS*) functions as a master determinant of axillary meristem formation, and loss-of-function mutants typically exhibit severe reductions in branching capacity [30,31]. In contrast, BRC1 acts as a central repressor of bud outgrowth and integrates multiple hormonal and environmental signals [32,33]. Together with other members of the GRAS (GIBBERELLIN INSENSITIVE, REPRESSOR OF GAI, and SCARECROW) transcription factor family, these genes form a regulatory framework linking meristem establishment, bud formation, and hormone responsiveness [34].

Although extensive studies have demonstrated that *LAS* genes play conserved roles in axillary meristem initiation and shoot branching [35], the mechanisms underlying *LAS*-mediated regulation of plant architecture in woody species remain largely unexplored [36]. In particular, whether LAS proteins function solely as developmental regulators or also participate in hormone-mediated modulation of branching remains unclear. GAs are critical regulators of plant architecture, but how GA homeostasis is integrated with axillary meristem development during branching remains poorly understood, especially in woody medicinal and ornamental plants.

In this study, we investigated the molecular basis of hormone-induced shoot branching in *Clerodendrum japonicum* by integrating physiological analysis, transcriptome sequencing, weighted gene co-expression network analysis (WGCNA), and functional characterization. We hypothesized that *CjLAS* acts as a regulatory hub linking axillary meristem development and GA homeostasis to control shoot branching. To test this hypothesis, we first characterized the effects of GA_3_ and 6-BA treatments on branch formation and identified branching-associated transcriptional networks. Subsequently, *CjLAS* was selected as a key candidate gene and its biological function was examined through ectopic overexpression in *Arabidopsis thaliana*. Furthermore, changes in GA metabolism and related gene expression were analyzed to explore the potential hormonal mechanism underlying *CjLAS*-mediated branching regulation. Our findings reveal an expanded role of LAS proteins in coordinating developmental and hormonal processes and provide new insights into the molecular regulation of shoot architecture in woody plants.

## 2. Results

### 2.1. Hormone Optimization and Temporal Dynamics of GA_3_/6-BA-Induced Branch Formation in C. japonicum

**Figure 1:**
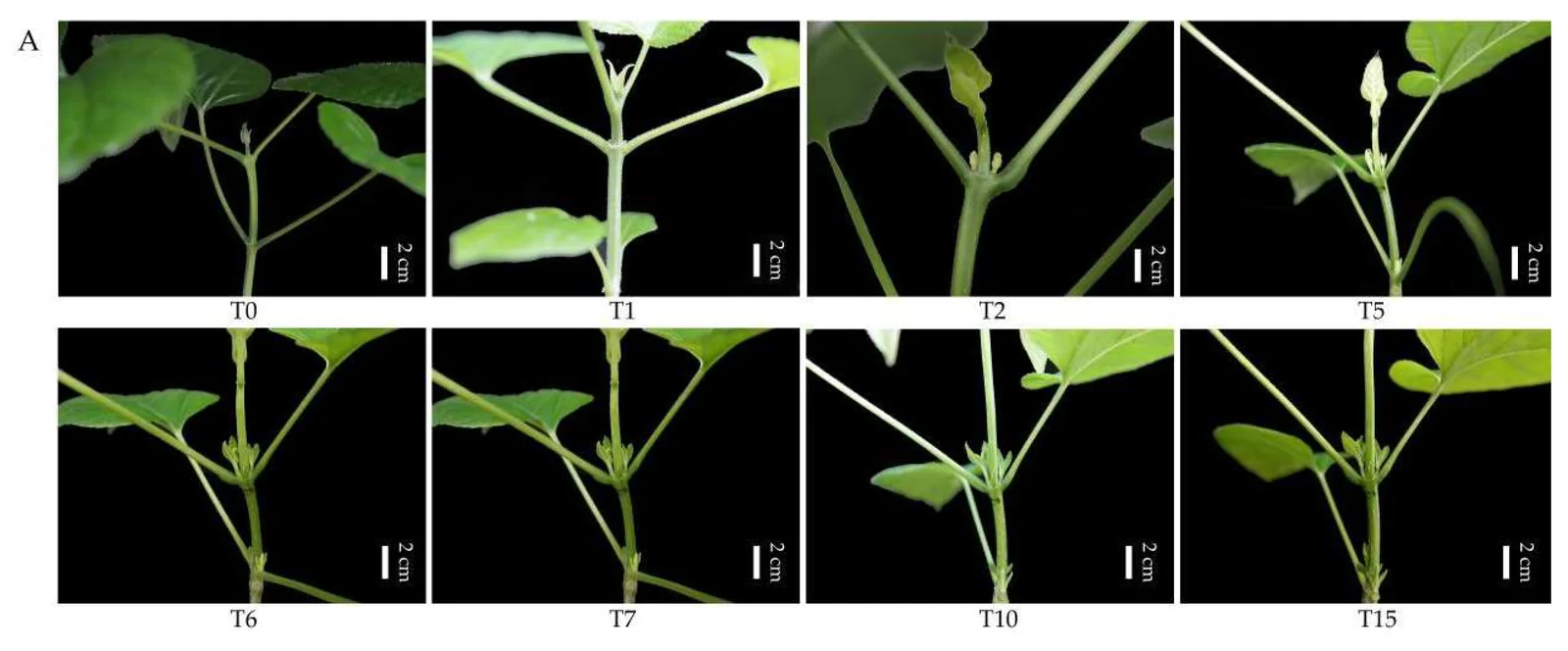

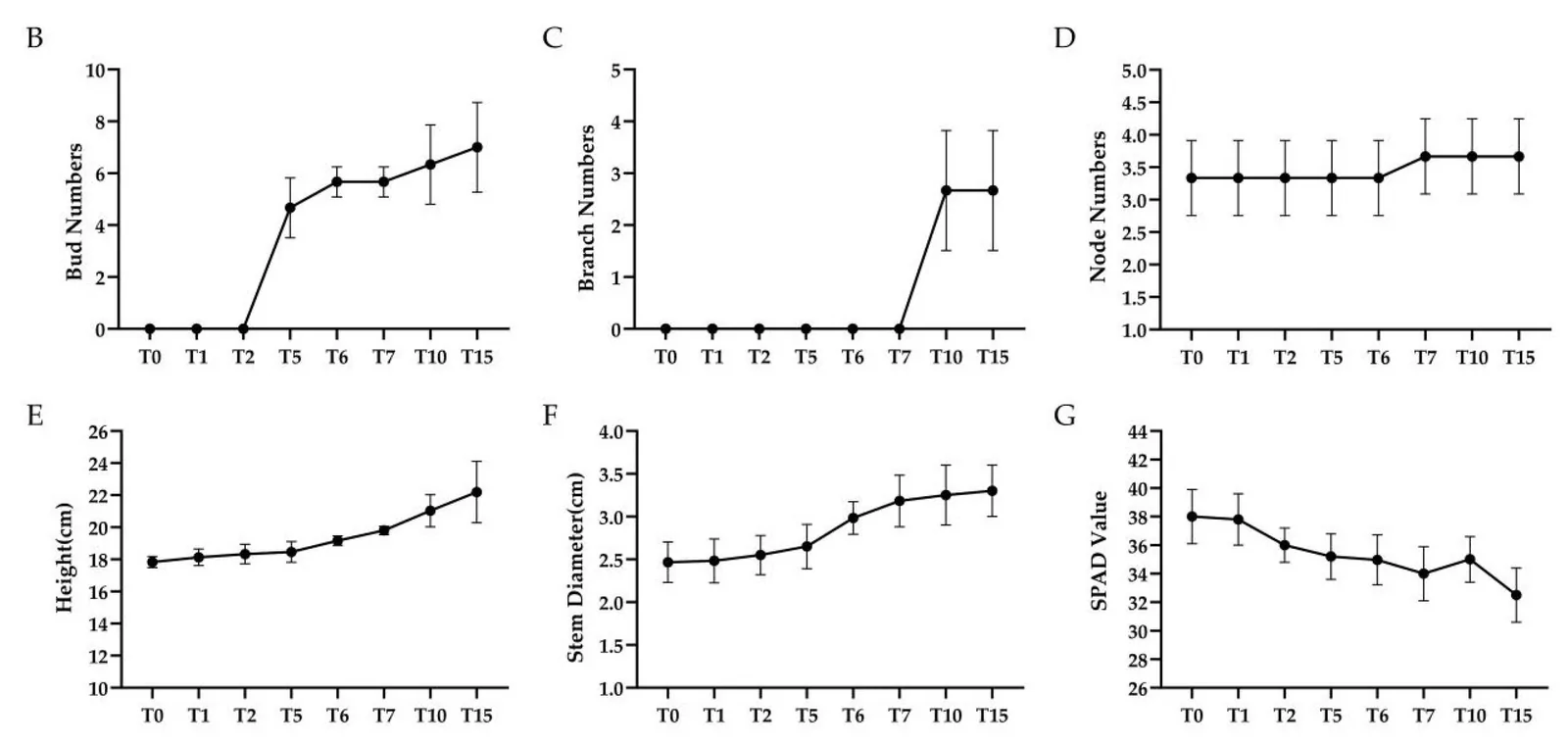
Dynamic changes in branching-related traits during exogenous hormone-induced axillary bud development in *C. japonicum*. (**A**) Phenotypic changes at 0, 1, 2, 5, 6, 7, 10, and 15 days after hormone treatment. (**B**–**G**) Changes in bud number, branch number, node number, plant height, stem diameter, and SPAD value during the induction process under the optimal hormone treatment (1500 mg L^−1^ total concentration of GA_3_ and 6-BA, with a 6-BA:GA_3_ ratio of 1:0.5). Data are presented as mean ± standard error (SE) from three biological replicates (*n* = 3).

To determine the optimal hormonal conditions for branch induction, *C. japonicum* plants were treated with various concentrations and combinations of GA_3_ and 6-benzyladenine (6-BA) (Supplementary Figure S1). Among the tested treatments, a concentration of 1500 mg L^−1^ at a 6-BA:GA_3_ ratio of 1:0.5 most effectively promoted bud number and branch formation; consequently, this formulation was selected for subsequent experiments (Figure 1A). No obvious differences were observed at early stages (T0–T2), while axillary bud formation became evident from T5 onwards under hormone treatments (Figure 1B). Bud number increased rapidly after T5, followed by a subsequent increase in branch formation at T10–T15 (Figure 1B,C). Node number remained relatively stable across all treatments (Figure 1D). Plant height and stem diameter showed gradual increases over time (Figure 1E,F), whereas SPAD values exhibited a slight decreasing trend during later stages (Figure 1G).

### 2.2. Transcriptomic Profiling Reveals Developmental Changes During Branch Induction Under Hormone Treatment

**Figure 2:**
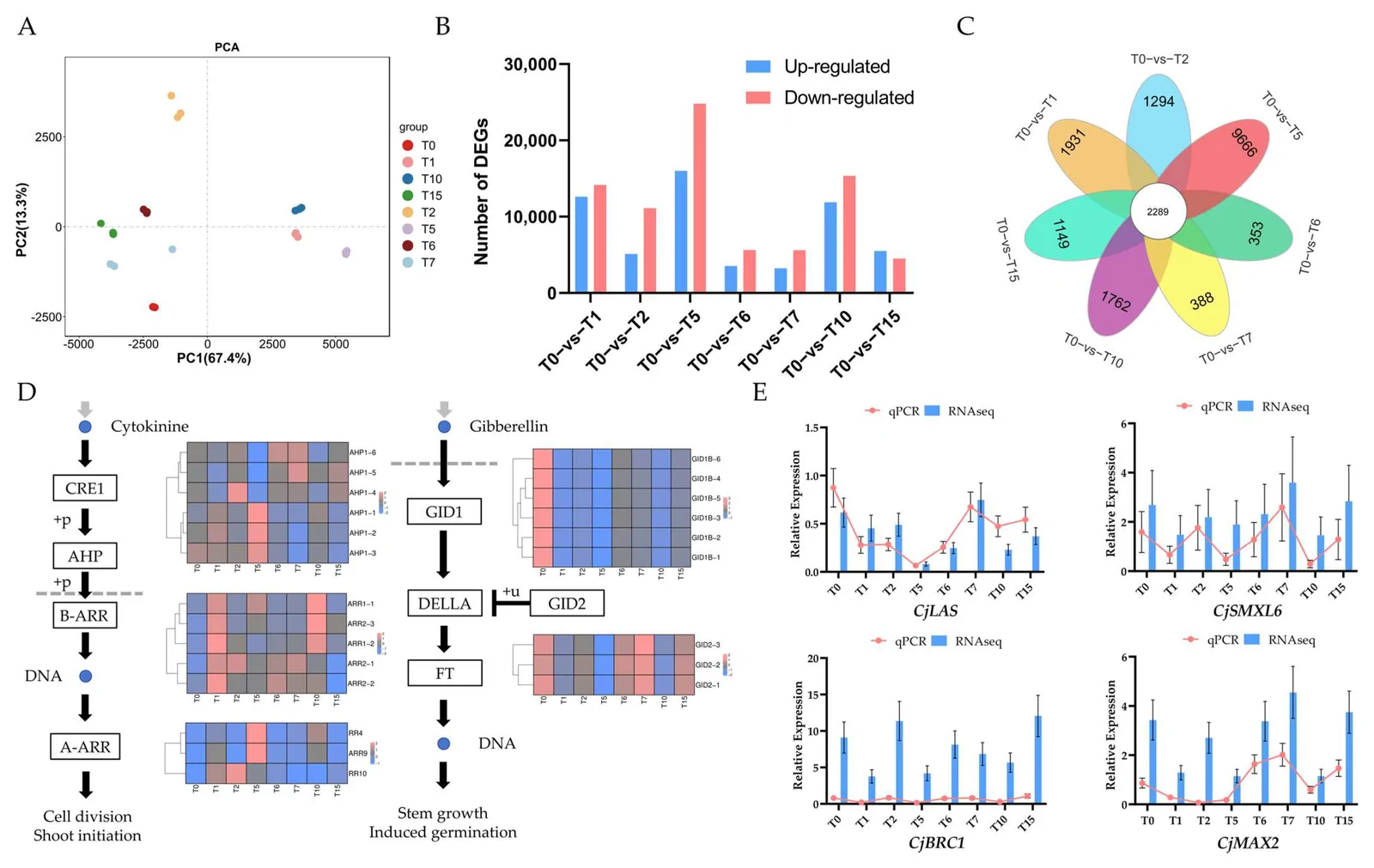
Transcriptomic analysis and identification of branch-related regulatory pathways during hormone-induced shoot branching in *C. japonicum*. (**A**) Principal component analysis (PCA) of transcriptome samples collected at different developmental stages. (**B**) Number of differentially expressed genes (DEGs) identified between T0 and subsequent developmental stages. (**C**) Venn diagram showing shared and stage-specific DEGs during branch induction. (**D**) Expression profiles of genes involved in cytokinin and GA signaling pathways during shoot branching. (**E**) Validation of RNA-seq data by RT-qPCR analysis of representative genes.

RNA-seq was performed using samples collected from eight developmental stages during branch induction. After quality filtering, approximately 58 Gb of clean bases were obtained from all transcriptome libraries, with Q30 values above 90% and high-quality sequencing data retained for subsequent analysis (Supplementary Table S2). In total, 137,206 expressed transcripts were detected, including 31,368 annotated transcripts in the full-length transcriptome reference and 105,838 novel transcripts. Principal component analysis (PCA) clearly separated samples from different developmental stages, whereas biological replicates clustered closely together, indicating the high reproducibility of the transcriptome dataset (Figure 2A). Differential expression analysis was subsequently performed using DESeq2 with T0 as the reference stage (|log_2_FC| ≥ 1, *p* ≤ 0.05). Large numbers of DEGs were identified throughout shoot branching (Figure 2B). The highest number of DEGs (40,823) was detected at T5 relative to T0, while downregulated genes predominated across most developmental stages, which suggested that branch induction involved substantial transcriptomic reorganization during developmental transition. Venn analysis further revealed both stage-specific and commonly regulated DEGs among developmental stages (Figure 2C).

GO and KEGG enrichment analyses were performed using 2289 representative DEGs. A total of 100 GO terms and 87 KEGG pathways were significantly enriched (Supplementary Figure S2). Among them, hormone-related biological processes and signaling pathways, including cytokinin- and GA-mediated signaling, plant hormone signal transduction, and MAPK signaling pathway, were prominently enriched, indicating that hormone signaling constitutes a major regulatory component during branch induction. At the gene level, 14 cytokinin-related genes and 8 GA-related genes showed differential expression across the sampled developmental stages under the selected hormone treatment (Figure 2D). Several type-B Arabidopsis response regulators (β-ARRs) were predominantly upregulated across developmental stages, whereas the GA receptor gene GID1 showed reduced transcript abundance. The expression patterns of four representative genes (*CjLAS*, *CjSMXL6*, *CjMAX2*, and *CjBRC1*) were further validated by RT-qPCR, and the results were highly consistent with the RNA-seq data (Figure 2E).

### 2.3. Co-Expression Network-Based Identification of Candidate Regulators of Branching

**Figure 3:**
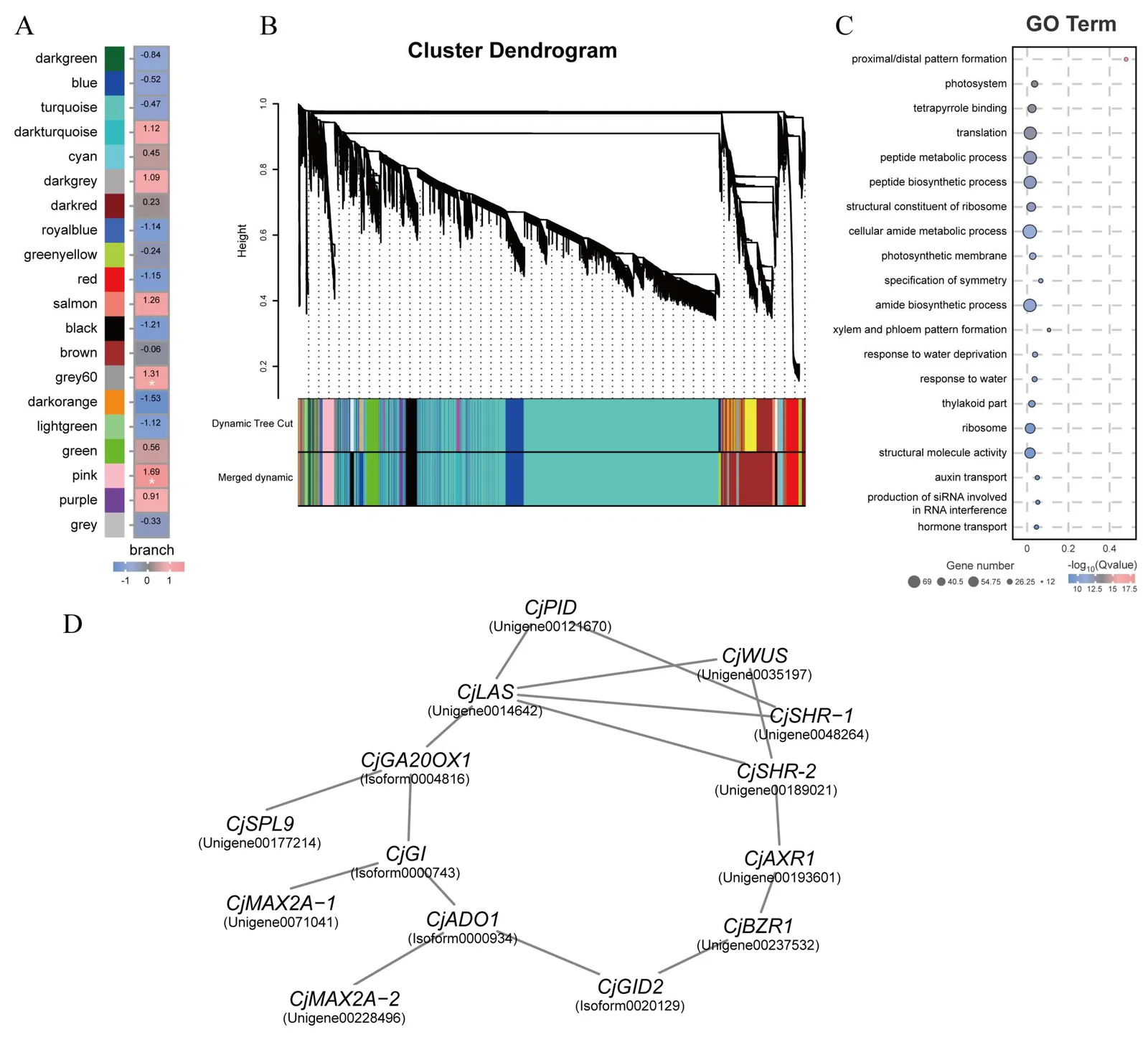
WGCNA of branch-related traits and identification of candidate regulatory modules. (**A**) Module–trait correlation heatmap showing relationships between gene co-expression modules and branch-related phenotypic traits. Asterisks indicate significant correlations (* *p* < 0.05). (**B**) Gene dendrogram and module assignment obtained from hierarchical clustering analysis. (**C**) Gene Ontology (GO) enrichment analysis of genes within the pink module, summarized as a circular enrichment plot. (**D**) Protein–protein interaction (PPI) network constructed for genes in the pink module, illustrating potential functional interactions among module genes.

WGCNA was performed to identify gene modules associated with shoot branching (Figure 3A,B). Among the identified modules, the pink module showed the strongest positive association with branch number and was therefore selected as a candidate module for further analysis. Functional enrichment analysis revealed that genes within this module were predominantly involved in developmental processes, reproductive bud development, and plant hormone signal transduction, suggesting an important role in branch formation (Figure 3C). To further explore functional relationships among genes within the pink module, a protein–protein interaction (PPI) network was constructed (Figure 3D), revealing a complex interaction network among module genes, with *CjLAS* connecting to multiple nodes involved in hormone signaling and meristem development, suggesting its potential role as a regulatory hub within the module.

Candidate genes associated with shoot branching were subsequently screened based on multiple criteria, including module membership, expression patterns consistent with developmental progression, functional annotation related to shoot development or meristem activity, and homologous relationships with previously characterized branching regulators in model plants. Based on these criteria, four candidate genes, namely *CjLAS*, *CjSHR*, *CjGI*, and *CjGA20ox1*, were identified for further analysis. Although *CjLAS* was not among the highest-connectivity hub genes in the pink module, its expression profile closely followed the module eigengene pattern. Phylogenetic analysis further indicated that *CjLAS* belongs to the GRAS transcription factor family and shows high similarity to LAS in Arabidopsis and MONOCULM1 (MOC1) in rice. Therefore, *CjLAS* was selected as a representative candidate gene for subsequent functional characterization.

### 2.4. Molecular Characterization of CjLAS

**Figure 4:**
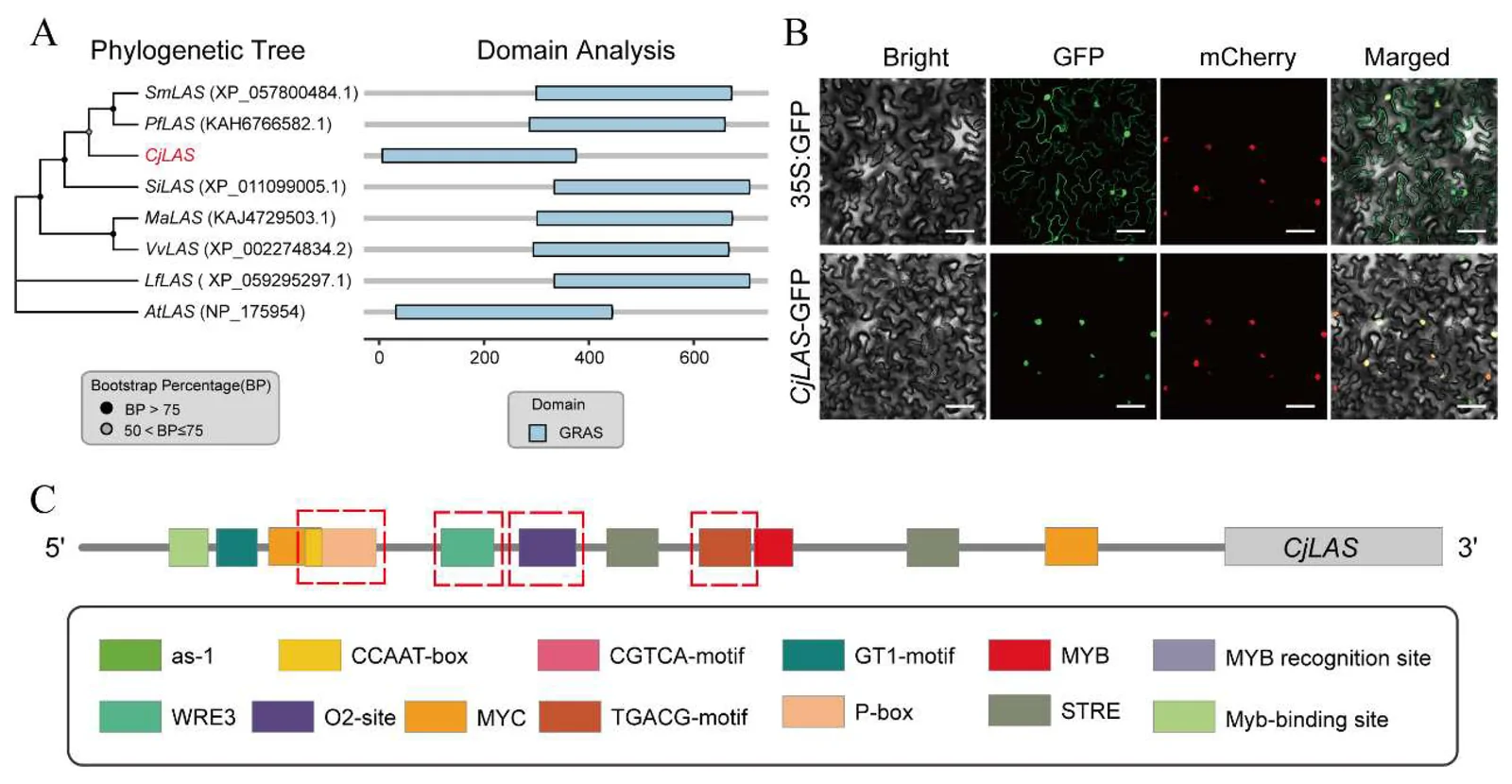
Structural features and promoter analysis of the branch regulatory factor *CjLAS.* (**A**) Phylogenetic analysis of *CjLAS* and its homologs from representative plant species, together with conserved domain architecture. (**B**) Subcellular localization of the CjLAS protein (Bar = 80 μm). (**C**) Distribution of cis-acting regulatory elements in the promoter region of *CjLAS*.

Phylogenetic analysis indicated that CjLAS clustered closely with LAS homologs from *Salvia miltiorrhiza* and *Perilla frutescens*, suggesting strong evolutionary conservation within Lamiaceae species (Figure 4A). Domain analysis further revealed that these proteins contained a conserved GRAS domain, consistent with their classification as members of the LAS subfamily of GRAS transcription factors. The subcellular localization of CjLAS was exclusively in the nucleus, overlapping with the nuclear localization signal (Figure 4B). This suggests that CjLAS functions as a transcription factor. To investigate potential upstream regulatory features, approximately 1007 bp of the promoter region upstream of the *CjLAS* start codon was isolated and analyzed. In addition to core promoter elements (TATA-box and CAAT-box), multiple hormone- and stress-responsive cis-elements were identified, including a GA-responsive P-box, methyl jasmonate-responsive elements, and light-responsive motifs (Figure 4C; Supplementary Figure S3; Supplementary Table S3).

### 2.5. Protein Interaction Network Associated with CjLAS

**Figure 5:**
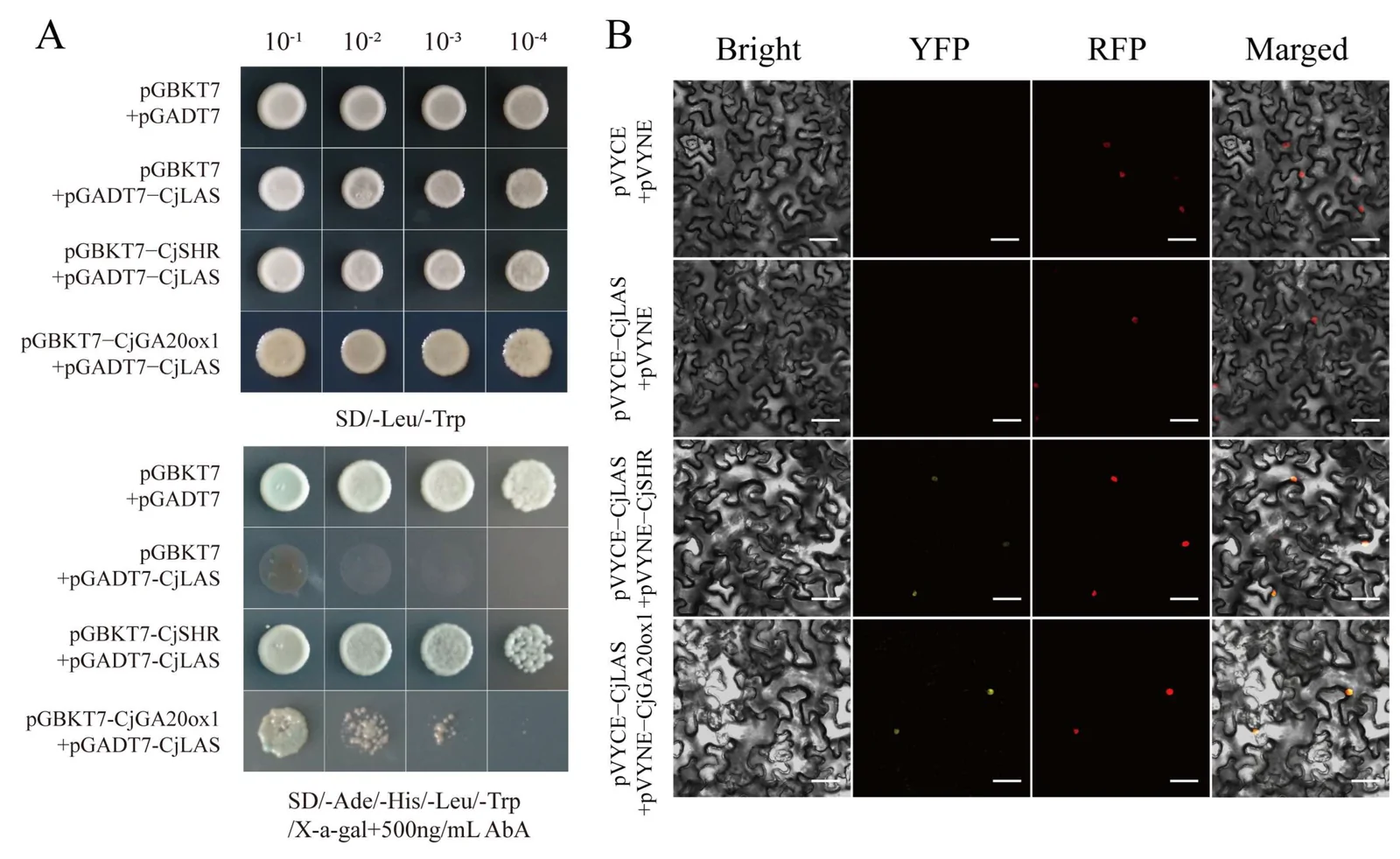
Protein–protein interaction analysis of CjLAS and its associated regulatory proteins. (**A**) Yeast two-hybrid (Y2H) assays validating protein–protein interactions involving CjLAS. (**B**) Bimolecular fluorescence complementation (BiFC) analysis showing interactions between CjLAS and candidate regulatory proteins in planta (Bar = 50 μm). YFP fluorescence, BiFC signal indicating protein–protein interaction; RFP fluorescence, H2B nuclear marker.

Systematic yeast two-hybrid pairwise interaction assays identified direct binary interactions between CjLAS and CjSHR, as well as between CjLAS and CjGA20ox1 (Figure 5A). To validate these interactions in planta, bimolecular fluorescence complementation (BiFC) assays were performed. Reconstituted YFP fluorescence with clear nuclear localization was observed in tobacco epidermal cells co-expressing CjLAS with CjSHR or CjGA20ox1, whereas no fluorescence signal was detected in the corresponding negative controls (Figure 5B). These results confirmed the physical interactions of CjLAS with both CjSHR and CjGA20ox1 in plant cells.

### 2.6. CjLAS Promotes Shoot Branching Through Conserved Developmental Function

**Figure 6:**
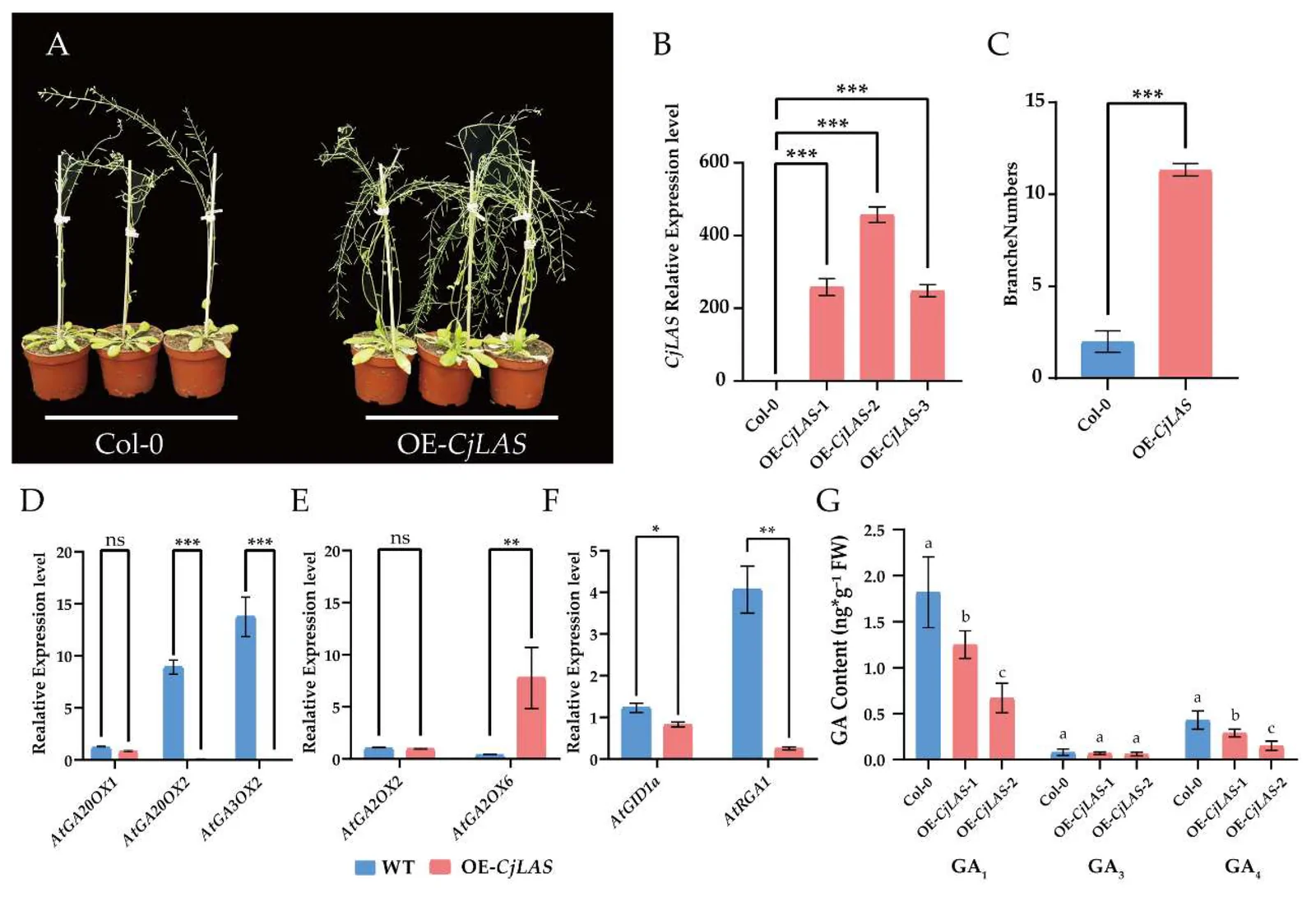
Overexpression of *CjLAS* promotes branching and alters GA-related gene expression in *Arabidopsis*. (**A**) Phenotypic comparison between wild-type (WT) and OE-*CjLAS* lines. (**B**) Relative expression level of *CjLAS* in WT and OE-*CjLAS* lines. (**C**) Comparison of branch number between WT and transgenic plants. (**D**) Expression analysis of genes involved in GA biosynthesis (*AtGA20ox1*, *AtGA20ox2*, *AtGA3ox2*), catabolism (*AtGA2ox2*, *AtGA2ox6*), and signaling (*AtGID1a, AtRGA1*). Data are presented as mean ± standard error (SE) (*n* = 3). For panels (**B**–**F**), statistical significance was determined using Student’s *t*-test: * *p* < 0.05; ** *p* < 0.01; *** *p* < 0.001; ns, not significant (*p* > 0.05). (**G**) Endogenous contents of bioactive GAs (GA_1_, GA_3_, and GA_4_) in wild-type (Col-0) and *CjLAS*-overexpressing *Arabidopsis* lines. GA contents were measured by LC–MS/MS in 14-day-old seedlings of Col-0, OE-*CjLAS*-1, and OE-*CjLAS*-2 lines. Data are presented as mean ± standard error (SE) from three independent biological replicates (*n* = 3 per genotype, with 10 seedlings pooled per replicate). Different letters indicate significant differences according to one-way ANOVA followed by Tukey’s HSD test (*p* < 0.05).

To investigate the biological function of *CjLAS* in shoot branching, a *CjLAS* overexpression construct was generated and introduced into Arabidopsis. Phenotypic analysis revealed a marked increase in branching capacity in OE-*CjLAS* lines (Figure 6A). The highest-expressing line exhibited approximately a five-fold increase in branch number compared with WT plants (Figure 6C), indicating a strong association between *CjLAS* expression and branch formation capacity. RT-qPCR analysis further demonstrated significantly elevated expression levels of *CjLAS* in transgenic lines compared with WT (Figure 6B).

To examine the effects of *CjLAS* overexpression on branch-related pathways, the expression of genes involved in GA biosynthesis, catabolism and signaling was analyzed. Compared with WT, the transcript levels of the GA biosynthetic genes *AtGA3ox2* and *AtGA20ox2* were significantly reduced in the overexpression lines, whereas *AtGA20ox1* showed no significant difference (Figure 6D). In contrast, expression of the GA catabolic gene *AtGA2ox6* was significantly increased (Figure 6E). The GA signaling genes *AtGID1a* and *AtRGA1* also exhibited lower transcript levels in the transgenic lines (Figure 6F).

To determine whether the transcriptional alterations in GA-related genes translated into changes in endogenous GA pools, we quantified the contents of bioactive GAs (GA_1_, GA_3_, and GA_4_) in OE-*CjLAS* lines and wild-type (WT) plants by liquid chromatography–tandem mass spectrometry (LC–MS/MS). As shown in Figure 6G, the OE lines exhibited significantly reduced levels of GA_1_ and GA_4_ compared with WT. Specifically, GA_1_ content decreased from 1.82 ± 0.23 ng g^−1^ FW in WT to 0.67 ± 0.09 ng g^−1^ FW in the OE-*CjLAS*-2 line (*p* < 0.01), and GA_4_ content declined from 0.43 ± 0.06 ng g^−1^ FW to 0.15 ± 0.03 ng g^−1^ FW (*p* < 0.01). In contrast, GA_3_ levels remained largely unchanged (WT: 0.08 ± 0.02 ng g^−1^ FW; OE-*CjLAS*-2: 0.06 ± 0.01 ng g^−1^ FW; *p* > 0.05). These results are consistent with the observed downregulation of GA biosynthetic genes (AtGA20ox2 and AtGA3ox2) and upregulation of the catabolic gene AtGA2ox6 in the OE lines (Figure 6D,E), collectively indicating that constitutive expression of *CjLAS* leads to a net reduction in bioactive GA content. This GA-deficient status is known to favor axillary meristem initiation and bud outgrowth, supporting the positive role of *CjLAS* in promoting branching (Figure 6A,C).

## 3. Discussion

### 3.1. Coordinated Cytokinin and GA Signaling Orchestrates Branch Induction

Branch formation is a developmental process involving two tightly coordinated stages: axillary meristem establishment and subsequent bud outgrowth [11,12]. These stages are regulated by multiple phytohormones that perform distinct yet interconnected functions [22]. CKs are widely recognized as positive regulators of axillary meristem initiation and bud formation, primarily through antagonizing apical dominance and suppressing branching inhibitors such as BRC1 [19,37]. In contrast, GAs are generally associated with cell elongation, meristem activity, and organ growth [35,38]. Consistent with these established functions, exogenous GA_3_ treatment in the present study markedly promoted stem elongation but exerted little influence on branch formation, whereas 6-BA significantly increased bud number while having a comparatively limited effect on subsequent branch elongation. Notably, simultaneous application of GA_3_ and 6-BA resulted in substantially greater branching than either hormone alone, demonstrating that the combined treatment produced a greater response.

These findings indicate that cytokinin and GA play distinct but complementary roles during branch induction. Cytokinin appears to primarily promote axillary meristem initiation, whereas GA contributes to subsequent bud growth and branch elongation. Their combined treatment effects support the concept that shoot branching is a continuous “meristem initiation–bud outgrowth” process coordinated by multiple hormonal pathways rather than by individual phytohormones acting independently [29,39]. Importantly, the clear developmental transition observed following hormone treatment also provided an ideal framework for dissecting the underlying molecular regulatory network through transcriptome analysis.

### 3.2. Transcriptome-Guided Identification of CjLAS as a Key Branching Regulator

Shoot branching is a complex quantitative trait controlled by coordinated interactions among multiple genes and signaling pathways [31]. Although differential expression analysis effectively captures genes responsive to developmental transitions, it often identifies thousands of transcriptionally altered genes, making it difficult to distinguish central regulators from downstream responses [40]. To address this limitation, WGCNA was employed to identify modules associated with branching-related phenotypes [41]. The pink module exhibited the strongest correlation with branch number and was significantly enriched in pathways associated with hormone signaling, meristem development, circadian regulation, and growth-related processes. Given the developmental nature of the time-course dataset, the pink module may capture both branching-associated and broader developmental signals. Therefore, candidate genes were prioritized by integrating co-expression relationships with their expression dynamics, functional annotation, and evolutionary evidence rather than by correlation with branch number alone. This analysis identified several candidate regulators, including *CjLAS*, *CjSHR*, *CjGA20ox1* and *CjGI*.

Among these candidates, *CjLAS* was selected for detailed characterization because LAS homologs have been established as indispensable regulators of axillary meristem formation in multiple plant species. Although *CjLAS* was not the highest-connectivity hub gene within the module, its expression pattern closely followed the module eigengene and was highly consistent with shoot branching progression. These observations emphasize that biologically important regulatory genes are not necessarily network hubs and highlight the value of combining co-expression analysis with functional annotation and evolutionary evidence for candidate gene prioritization.

### 3.3. CjLAS Coordinates Axillary Meristem Establishment Through GA-Associated Regulation

LATERAL SUPPRESSOR (LAS) is one of the most conserved GRAS transcription factors controlling axillary meristem formation. Loss-of-function mutations of LAS in *Arabidopsis* abolish vegetative lateral shoot formation, whereas mutation of its rice ortholog MOC1 dramatically reduces tiller production, demonstrating that LAS family proteins play evolutionarily conserved roles in branching [13,30]. Consistent with these previous findings, constitutive expression of *CjLAS* in *Arabidopsis* significantly increased branch number, confirming that its positive regulatory function is largely conserved across species.

Our results further extend the current understanding of LAS function. In addition to its conserved role in axillary meristem development, several lines of evidence suggest that *CjLAS* participates in GA-associated regulatory processes. Promoter analysis identified a GA-responsive P-box together with multiple hormone-responsive cis-elements, indicating that *CjLAS* itself may respond to diverse hormonal cues. Furthermore, overexpression of *CjLAS* resulted in coordinated transcriptional changes affecting multiple components of the GA pathway. Expression of the biosynthetic genes GA20ox2 and GA3ox2 decreased significantly, whereas the GA catabolic gene GA2ox6 was induced. Simultaneously, key signaling components, including GID1a and RGA1, also exhibited reduced transcript abundance. In addition to these transcriptional changes, we directly quantified endogenous bioactive GA contents to determine whether the observed gene expression alterations were reflected at the metabolite level. Quantification of endogenous bioactive GAs revealed that *CjLAS* overexpression led to a significant reduction in GA_1_ and GA_4_ contents (Supplementary Figure S4), with the high-expression line OE-*CjLAS*-2 exhibiting the most pronounced decreases (GA_1_: from 1.82 ± 0.23 to 0.67 ± 0.09 ng g^−1^ FW; GA_4_: from 0.43 ± 0.06 to 0.15 ± 0.03 ng g^−1^ FW). These biochemical data provide direct evidence that *CjLAS* may influence GA homeostasis through transcriptional and protein-level regulatory mechanisms. The consistency between transcriptomic changes (downregulation of biosynthetic genes, upregulation of catabolic genes) and metabolic outcomes (decreased GA_1_/GA_4_ pools) strongly supports the notion that *CjLAS* decreases GA activity associated with branching promotion. Given that reduced GA activity has been shown to promote axillary meristem maintenance and bud outgrowth in multiple species, these findings mechanistically link *CjLAS*-mediated GA homeostasis to the enhanced branching phenotype observed in transgenic plants (Figure 5A,C). Similar regulatory relationships have been reported in several plant species, where localized reductions in GA activity promote axillary meristem maintenance [29] and branching while restricting excessive stem elongation [42].

Protein interaction analyses provided additional evidence supporting the multifunctional role of CjLAS. Both yeast two-hybrid and BiFC assays demonstrated direct interactions between CjLAS and CjSHR, as well as between CjLAS and CjGA20ox1. SHR proteins are well-known regulators of stem cell maintenance and meristem organization [43,44], whereas GA20ox proteins catalyze key rate-limiting steps in GA biosynthesis [45,46]. The physical association of CjLAS with these proteins suggests that CjLAS may coordinate developmental regulation with hormone metabolism through both transcriptional and protein-level mechanisms. In addition, the predicted interaction with CjGI, a central regulator of circadian rhythm and photoperiodic signaling [47,48], further implies that CjLAS may integrate developmental, hormonal, and environmental signals during branch formation. One possible explanation is that *CjLAS* does not directly regulate GA metabolism itself but rather fine-tunes GA homeostasis through interactions with developmental regulators and GA biosynthetic proteins, thereby coordinating the transition from axillary meristem establishment to subsequent bud outgrowth. Collectively, these findings indicate that *CjLAS* is closely associated with GA-mediated shoot branching and prompted us to further consider its broader regulatory role.

### 3.4. An Expanded Functional Framework of CjLAS-Mediated Branch Regulation

Previous studies have primarily described LAS as a developmental regulator required for axillary meristem initiation. By integrating physiological, transcriptomic, and molecular evidence, our study suggests that the regulatory role of LAS may extend beyond meristem establishment. Rather than functioning solely as a determinant of axillary meristem identity, *CjLAS* appears to coordinate developmental programs with GA-associated regulatory processes during branch formation. This expanded functional framework is supported by the hormone-responsive promoter architecture of *CjLAS*, its coordinated effects on GA-related gene expression, and its interactions with proteins involved in meristem organization and GA metabolism.

Notably, the reduction in endogenous bioactive GA levels following exogenous GA_3_ treatment (as inferred from *Arabidopsis* data) may involve a classical feedback regulation mechanism. In many species, increased GA signalling suppresses the transcription of GA biosynthesis genes (*GA20ox*, *GA3ox*) and activates catabolic genes (*GA2ox*), forming a homeostatic loop. In our study, the downregulation of *AtGA20ox2* and *AtGA3ox2* and upregulation of *AtGA2ox6* in *CjLAS*-overexpressing lines support such a feedback model, which may also operate in *C. japonicum*. This feedback could create a low-GA state favorable for axillary bud outgrowth, counteracting the initial exogenous GA pulse. Ideally, quantification of endogenous GA levels in *C. japonicum* axillary buds after hormone treatment would further substantiate the regulatory role of *CjLAS*. However, due to limited sample availability, this was not feasible in the current study and should be addressed in future investigations.

Although the direct downstream targets of *CjLAS* remain to be identified, our findings provide a conceptual framework for understanding how developmental regulators and hormone signaling are integrated during branching in woody plants. Future studies using yeast one-hybrid (Y1H), electrophoretic mobility shift assays (EMSA), and promoter–reporter assays will be necessary to determine whether CjLAS directly binds to and regulates specific GA-related promoters.

### 3.5. Biological Significance and Potential Application of CjLAS-Mediated Branching Regulation

Shoot branching is a complex developmental trait that determines plant architecture, reproductive performance, and biomass allocation. Although *LAS* genes have been widely recognized as key regulators of axillary meristem initiation, their functional roles in woody plants remain insufficiently characterized. In this study, the identification and functional characterization of *CjLAS* provide evidence that LAS-mediated regulation extends beyond meristem establishment and involves coordination with endogenous GA homeostasis. This finding highlights a conserved yet previously underexplored mechanism through which developmental regulators and hormone metabolism interact to shape plant architecture.

From an applied perspective, understanding the molecular basis of *CjLAS*-mediated branching regulation may provide valuable genetic resources for improving plant architecture in ornamental and medicinal species. Manipulation of branching traits through endogenous regulatory pathways could contribute to the optimization of plant form, biomass distribution, and cultivation practices. However, further studies using loss-of-function approaches and tissue-specific analyses are required to determine the precise regulatory mechanisms of *CjLAS* under field conditions.

## 4. Materials and Methods

### 4.1. Plant Materials and Growth Conditions

Rooted cuttings of *C. japonicum* were obtained from the nursery of the Guangdong Academy of Forestry Sciences (Guangzhou, China; 23°11′59″ N, 113°22′11″ E). Uniform and healthy plants (15–20 cm in height with at least three stem nodes) were selected as the experimental materials. Arabidopsis ecotype Columbia-0 (Col-0) was used for stable genetic transformation, whereas *Nicotiana benthamiana* was used for transient expression assays, including subcellular localization and bimolecular fluorescence complementation (BiFC) analyses.

### 4.2. Exogenous Hormone Treatments

Gibberellic acid (GA_3_) and 6-benzyladenine (6-BA) were administered either alone or simultaneously to evaluate the impact of exogenous phytohormones on the branching patterns of *C. japonicum*. Phytohormone solutions were prepared at three concentrations (100, 1500, and 3000 mg L^−1^) and mixed at GA_3_:6-BA ratios of 0.5:1, 1:0.5, and 1:1 for the combined treatments. Plants sprayed with sterile distilled water functioned as the control group (CK). All treatments were administered via a single foliar application before axillary buds emerged. Phenotypic observations and sample collection were performed at 0, 1, 2, 5, 6, 7, 10, and 15 days after treatment (DAT). Plant height, stem diameter, node number, leaf number, bud number, and branch number were recorded. Lateral shoots exceeding 0.5 cm in length were defined as branches, whereas axillary structures longer than 2 mm were considered buds.

### 4.3. RNA Extraction, Library Preparation, and Sequencing

Total RNA was extracted from axillary buds collected at seven sampling points (0, 1, 2, 5, 6, 7, 10, and 15 DAT), with three independent biological replicates for each time point. RNA extraction was performed following the protocol provided in the plant RNAexr Plant Plus Kit (Huayueyang, Beijing, China). Briefly, tissues were ground into a fine powder in liquid nitrogen and homogenized in the lysis buffer. After centrifugation to clear cellular debris, the supernatant was mixed with ethanol to precipitate RNA and subsequently transferred to a spin column for selective binding. The column was washed twice with wash buffers to remove proteins and contaminants, dried by centrifugation, and the high-purity RNA was eluted in RNase-free water. Samples with an RNA integrity number (RIN) ≥ 8.0 were outsourced to Gene Denovo Biotechnology Co., Ltd. (Guangzhou, China) for the next processing step, including the purification of RNA, reverse transcription, construction of the library, and subsequent sequencing.

RT-qPCR was performed using a 2× RealStar Green Fast Mixture with ROX II (GenStar, Beijing, China), and relative expression levels were calculated using the 2^−ΔΔCt^ method with *CjUBQ* as the internal reference gene. All primers utilized for RT-qPCR are given in Supplementary Table S1. Three biological replicates were included for every RT-qPCR reaction.

### 4.4. Transcriptomic Analyses

High-quality clean reads were obtained by removing adaptor sequences, low-quality reads, and ambiguous bases from the raw sequencing data [49]. The clean reads were aligned to the *C. japonicum* full-length transcriptome reference dataset (National Genomics Data Center, BioProject: CRA005391) using HISAT2. Sequences that matched the existing reference transcripts were classified as annotated transcripts. Unmatched sequences were subsequently assembled and classified as novel transcripts. The resulting annotated and novel transcript sequences were incorporated into the transcriptome dataset for downstream expression and functional analyses. StringTie was used to assemble transcripts and estimate transcript abundance. FPKM values were calculated for expression profiling and visualization. For differential expression analysis, the raw gene-level integer count matrix was used as input for DESeq2 (v1.34.0). Genes with insufficient read counts were filtered before analysis. DESeq2 normalization was performed using the median-of-ratios method [50]. Differential expression was evaluated using the appropriate stage-versus-T0 contrasts, with |log_2_ (fold change)| ≥ 1 and Benjamini–Hochberg adjusted *p* ≤ 0.05 used as the significance criteria [38,50,51].

Differentially expressed genes (DEGs) were identified by pairwise comparison between the reference stage T0 and each of the seven subsequent developmental stages (T1, T2, T5, T6, T7, T10, and T15). Venn analysis was subsequently used to identify genes commonly differentially expressed across all seven comparisons, yielding 2289 common differentially expressed genes (DEGs), which were used for downstream functional enrichment analysis. GO and KEGG enrichment analyses were performed using a hypergeometric test, with all expressed genes detected in the transcriptome dataset used as the background gene universe. *p* values were corrected for multiple testing using the false discovery rate (FDR) method, and terms or pathways with FDR < 0.05 were considered significantly enriched. The raw sequencing data have been deposited in the CNCB Sequence Read Archive (SRA) under accession number PRJCA019175.

### 4.5. WGCNA

WGCNA was performed on the gene expression dataset to construct co-expression modules associated with branching-related traits in *C. japonicum* [52]. Gene expression levels were represented by FPKM values and transformed using log_2_ (FPKM + 1). Genes with a variance < 1 across all samples were removed, resulting in 24,499 genes retained for network construction. A signed weighted co-expression network was constructed using a soft-thresholding power of β = 8, which achieved a scale-free topology fit index of R^2^ = 0.85. The minimum module size was set to 30 genes, and modules with highly similar eigengene profiles were merged using a merge threshold of 0.25. Module eigengenes were correlated with branching-related phenotypic traits using Pearson correlation analysis. Multiple testing was controlled using the Bonferroni correction, and module–trait associations with adjusted *p* < 0.05 were considered significant. Gene-level module membership was evaluated based on the correlation between individual gene expression profiles and the corresponding module eigengene, and genes with module membership > 0.7 were considered strongly associated with their respective modules. The protein–protein interaction network was predicted using the STRING database (v11.5) based on orthology to *Arabidopsis thaliana*, with a confidence score ≥ 0.7. The network was visualized in Cytoscape (v3.8.2) [53].

### 4.6. Identification and Analysis of CjLAS

The LAS domain profile obtained from the Pfam database was employed to scan the transcriptome databases. After candidate gene screening, candidate genes encoding GRAS domains were confirmed using Pfam and the CDD-Search on NCBI, and named *CjLAS*. For phylogenetic analysis, the deduced amino acid sequence of CjLAS was aligned with LAS/MOC1-related protein sequences from representative plant species using MUSCLE implemented in MEGA 11. The best-fitting amino acid substitution model was selected using the Model Selection function in MEGA, and the JTT+G model was identified as the best-fit based on the Bayesian Information Criterion (BIC). A maximum-likelihood (ML) phylogenetic tree was subsequently inferred under the JTT+G model, with 1000 bootstrap replicates to evaluate branch support. The architectural features of conserved domains were characterized using the SMART and Pfam databases.

### 4.7. Subcellular Localization

Subcellular localization of *CjLAS* was carried out according to Tague’s method [54]. The full-length coding sequence (CDS) of *CjLAS* was amplified through PCR utilizing High-Fidelity DNA Polymerase (Takara Bio, Kusatsu, Japan) with gene-specific primers (Supplementary Table S1). The purified PCR product was cloned into the pCAMBIA1300 vector (expression cassette: 35S::CjLAS-EGFP). The *CjLAS*-GFP recombinant construct and the empty-vector control were introduced into *Agrobacterium tumefaciens* GV3103 and transiently expressed in *Nicotiana benthamiana* leaves by Agrobacterium-mediated infiltration. GFP Fluorescence signals were observed at approximately 72 h after infiltration using a laser scanning confocal microscope (Olympus FV1000, Tokyo, Japan), with an excitation wavelength of 488–550 nm.

### 4.8. Overexpression in Arabidopsis thaliana

Gene-specific primers were designed based on the coding sequence (CDS) of *CjLAS* (Supplementary Table S1). The full-length CDS was amplified using a high-fidelity DNA polymerase (Takara Bio, Kusatsu, Japan) and subsequently cloned into the pGreen-C1 expression vector under the control of the CaMV 35S promoter. Recombinant constructs were verified by Sanger sequencing. The validated plasmid was introduced into Agrobacterium tumefaciens strain GV3101 and transformed into Arabidopsis (Col-0) using the floral-dip method. Transgenic plants were selected on Basta-containing medium, and homozygous T3/T4 lines were obtained through successive generations of selection. Expression levels of *CjLAS* in transgenic plants were analyzed by RT-qPCR as described below.

### 4.9. RT-qPCR Analysis

To evaluate downstream gene expression in *CjLAS*-overexpressing lines, the transcript levels of genes involved in GA biosynthesis, catabolism, and signaling pathways were quantified by RT-qPCR. Target genes included *AtGA20ox1*, *AtGA20ox2*, *AtGA3ox2*, *AtGA2ox2*, *AtGA2ox6*, *AtGID1a* and *AtRGA1* (Supplementary Table S1). RT-qPCR was performed using a 2× RealStar Green Fast Mixture with ROX II (GenStar Biotechnology, Beijing, China), and relative expression levels were calculated using the 2^−ΔΔCt^ method with ACTIN as the internal reference gene.

### 4.10. Quantification of Endogenous GA Contents

Endogenous GA_1_, GA_3_, and GA_4_ contents were measured in 14-day-old seedlings of wild-type (WT) and OE-*CjLAS Arabidopsis* lines (OE-*CjLAS*-1 and OE-*CjLAS*-2). Approximately 0.5 g of fresh tissue per sample was ground in liquid nitrogen and extracted with 80% (*v*/*v*) methanol containing 0.1% (*v*/*v*) formic acid at 4 °C overnight. Deuterated internal standards ([^2^H_2_]-GA_1_, [^2^H_2_]-GA_3_, and [^2^H_2_]-GA_4_, OlChemIm Ltd., Olomouc, Czech Republic) were added to each sample prior to extraction for quantification. After centrifugation at 12,000 rpm for 10 min, the supernatant was purified using Oasis HLB solid-phase extraction columns (Waters, Milford, MA, USA) and analyzed by ultra-performance liquid chromatography–tandem mass spectrometry (UPLC–MS/MS) on a Waters ACQUITY UPLC system coupled with a Xevo TQ-S mass spectrometer (Waters, USA). GA contents were calculated based on the ratio of peak areas of endogenous compounds to their respective internal standards and expressed as nanograms per gram fresh weight (ng g^−1^ FW). Three independent biological replicates were performed for each genotype.

### 4.11. Promoter Cis-Regulatory Element Analysis

The promoter region of *CjLAS* was isolated using the GenomeWalker™ 2 Universal Kit (Takara, Japan) according to the manufacturer’s instructions. Genomic DNA was extracted from *Clerodendrum japonicum* leaves and used to construct GenomeWalker libraries. Gene-specific primers were designed based on the *CjLAS* sequence for nested PCR amplification (Supplementary Table S1). The amplified promoter fragments were cloned and sequenced for verification. The obtained promoter sequence (1007 bp upstream of the start codon) was analyzed for cis-acting regulatory elements using the PlantCARE database.

### 4.12. Yeast Two-Hybrid (Y2H) Assays

Y2H assays were performed using the Matchmaker Gold system in yeast strain AH109. The coding sequences of *CjLAS* were recombined into the pGADT7 vector, while *CjSHR* and *CjGA20ox1* were recombined into the pGBKT7 vector (Supplementary Table S1). Yeast transformants were selected on SD/-Leu/-Trp medium and interaction assays were conducted on SD/-Ade/-His/-Leu/-Trp medium supplemented with 3-AT.

### 4.13. Bimolecular Fluorescence Complementation (BiFC) Assays

For bimolecular fluorescence complementation (BiFC) assays, the coding sequences of *CjSHR* and *CjGA20ox1* were cloned into the pC1300-mVYNE vector, while *CjLAS* was cloned into the pC2300-mVYCE vector without stop codons (Supplementary Table S1). The constructs were introduced into *Agrobacterium tumefaciens* strain GV3101 and co-infiltrated into *Nicotiana benthamiana* leaves together with the nuclear marker H2B-mCherry. Empty vector combinations were used as negative controls. Fluorescence signals were observed 72 h after infiltration using a laser scanning confocal microscope (Olympus FV1000, Tokyo, Japan).

### 4.14. Statistical Analysis

All experiments were conducted with at least three biological replicates. Statistical analyses were performed using IBM SPSS Statistics 25.0 (IBM Corp., Armonk, NY, USA). Three independent plants were used as biological replicates for each experimental group, and the biological replicate was considered the experimental unit. Data are presented as mean ± standard error (SE). For comparisons between two groups, Student’s *t*-test was performed. For comparisons among multiple groups, one-way analysis of variance (ANOVA) followed by Tukey’s honestly significant difference (HSD) test was performed. Statistical significance was determined at *p* < 0.05, while *p* < 0.01 was considered highly significant.

## 5. Conclusions

**Figure 7:**
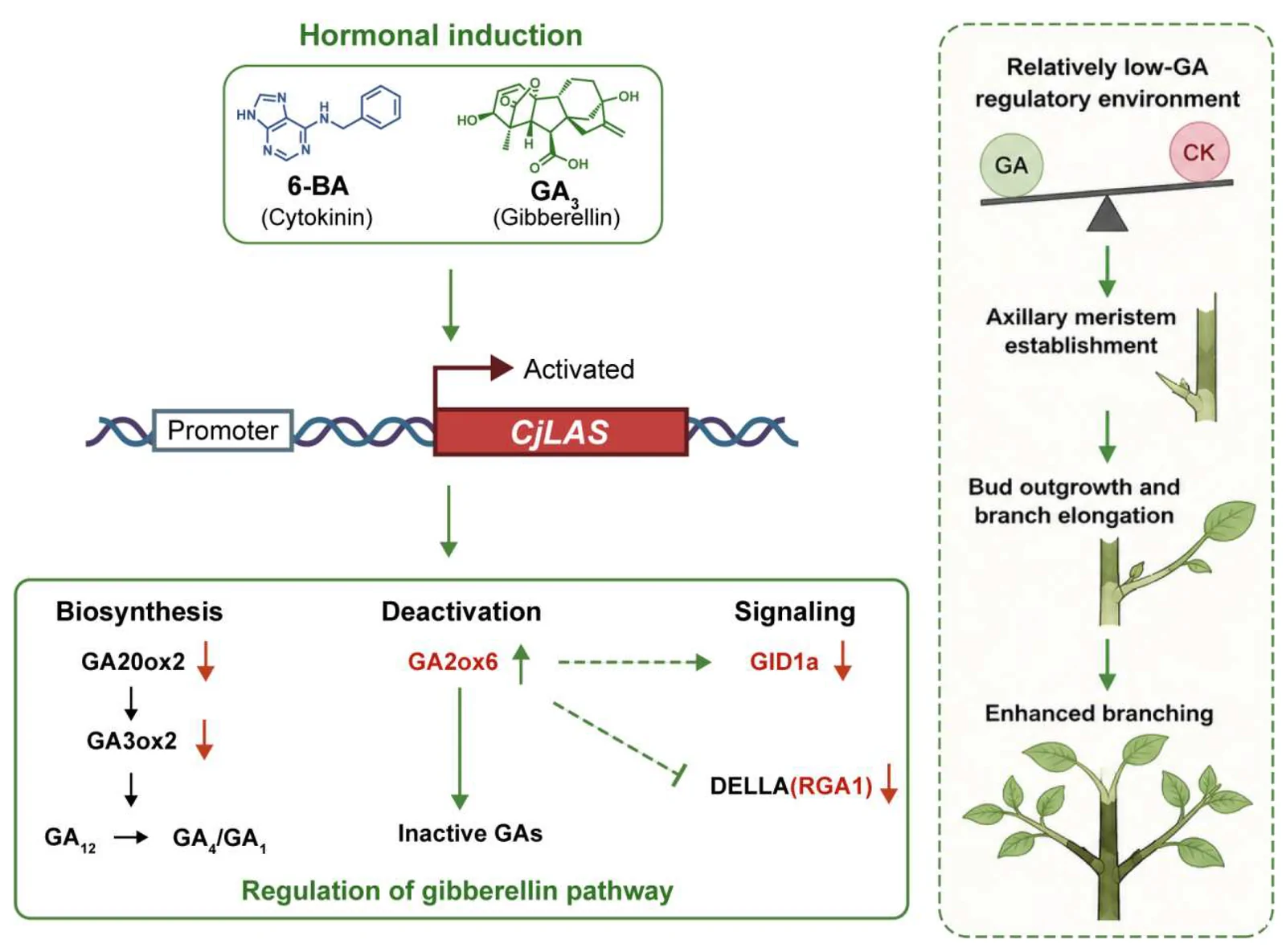
A proposed working model for the combined treatment regulation of axillary bud development and shoot branching by GA_3_ and 6-BA in *C. japonicum*.

This study elucidated the hormonal and molecular regulatory mechanisms underlying branch formation in *C. japonicum* (Figure 7). Combined application of 6-BA and GA_3_ effectively enhanced branching by promoting both axillary meristem initiation and subsequent bud outgrowth. Transcriptome analysis integrated with weighted gene co-expression network analysis identified *CjLAS* as a key regulator associated with shoot branching. Functional validation demonstrated that overexpression of *CjLAS* promoted branching and was associated with reduced expression of GA biosynthetic genes, increased expression of the GA catabolic gene AtGA2ox6, and reduced endogenous GA1 and GA4 contents. These findings suggest that *CjLAS* contributes to branching regulation through adjustment of GA homeostasis. The reduced GA biosynthetic activity may represent, at least in part, a homeostatic feedback response to altered GA status, resulting in a reduction in bioactive GA levels, which may favor branching. Protein interaction assays further revealed that CjLAS interacts with CjSHR and CjGA20ox1, suggesting the involvement of a *CjLAS*-mediated regulatory module linking axillary meristem development with GA-dependent branch growth. Collectively, these findings establish *CjLAS* as a regulatory component connecting axillary meristem development with GA homeostasis during shoot branching. This study expands the functional landscape of LAS proteins in woody plants and provides insights into how developmental regulators coordinate hormonal states to shape plant architecture.

Combined application of GA_3_ and 6-BA promotes branch formation by activating hormone-responsive regulatory networks, in which *CjLAS* functions as a key regulatory component. *CjLAS* overexpression was associated with transcriptional changes in genes involved in GA biosynthesis, catabolism, and signaling, thereby contributing to a relatively reduced GA activity state. This hormonal adjustment facilitates axillary meristem establishment and subsequent bud outgrowth, ultimately promoting shoot branching in *C. japonicum*.

## Data Availability

The raw RNA-seq data generated in this study have been deposited in the CNCB Sequence Read Archive (SRA) under accession number PRJCA019175.

## Supplementary Materials

The following supporting information can be downloaded at: https://www.mdpi.com/article/10.3390/plants15182827/s1, Supplementary Figure S1: Combined treatment regulation of branching traits by GA_3_ and 6-BA in *Clerodendrum japonicum*; Supplementary Figure S2: Functional enrichment analysis of DEGs during hormone-induced shoot branching based on GO and KEGG pathways; Supplementary Figure S3: Promoter sequence of *CjLAS* in *C. japonicum*; Supplementary Table S1: Primer information; Supplementary Table S2: Quality control analysis results; Supplementary Table S3: Cis-acting regulatory elements identified in the promoter region of *CjLAS*.
