# Supplementary material for: Integration of Axillary Meristem Development and Gibberellin Homeostasis by CjLAS Regulates Shoot Branching in Clerodendrum japonicum
Source: Plants (Basel). 2026 Sep 15;15(18):2827. doi: 10.3390/plants15182827 (PMC13610545; doi:10.3390/plants15182827)
Supplement: Supplementary file 1 [file plants-15-02827-s001.zip › Table S3.pdf]

Supplementary Table S3. Cis-acting regulatory elements identified in the promoter region of *CjLAS*.

Element type	Motif name	Motif（5′-3′）	Position (bp)	Strand	Strand	Function
Core promoter	CAAT-box	CCAAT	375	5	-	common cis-acting element in promoter and enhancer regions
		CAAT	443	4	-	
		CCAAT	451	5	-	
		CAAAT	471	5	-	
		CAAT	567	4	-	
		CAAT	675	4	-	
		CAAT	695	4	+	
		CAAT	786	4	+	
	TATA-box	TATAA	179	5	-	core promoter element around -30 of transcription start
		TATA	180	4	+	
		TATA	494	4	+	
		TATA	749	4	-	
		TATA	759	4	-	
		TACAAAA	772	7	+	
		TAAAGATT	787	8	-	
		TATAA	1000	5	-	
		TATA	1001	4	-	
Light regulatory	GT1-motif	GGTTAA	138	6	+	light responsive element
		GGTTAA	140	6	-	
Hormone response		CCTTTTG	231	7	-	gibberellin-responsive element
	CGTCA-motif	CGTCA	561	5	+	cis-acting regulatory element involved in the MeJA-responsiveness
	TGACG-motif	TGACG	561	5	-	
Others	as-1	TGACG	561	5	-	-
	CCAAT-box	CAACGG	216	6	+	MYBHv1 binding site
	MYB	CAACAG	97	6	+	-
		CAACCA	609	6	+	-
	MYB recognition site	CCGTTG	216	6	-	-
	Myb-binding site	CAACAG	97	6	+	-
	MYC	CATGTG	184	6	+	-
		CATGTG	864	6	-	-
	WRE3	CCACCT	335	6	+	-

Element type	Motif name	Motif（5′-3′）	Position (bp)	Strand	Strand	Function
	STRE	AGGGG	480	5	+	-
		AGGGG	743	5	+	-
	O2-site	GATGATGT GG	403	10	+	cis-acting regulatory element involved in zein metabolism regulation
	Unnamed__2	CCCCGG	309	6	+	-
	Unnamed__4	CTCC	116	4	+	-
		CTCC	132	4	-	-
		CTCC	165	4	+	-
		CTCC	272	4	+	-
		CTCC	321	4	-	-
		CTCC	387	4	-	-
		CTCC	418	4	+	-
