# Supplementary material for: Integration of Axillary Meristem Development and Gibberellin Homeostasis by CjLAS Regulates Shoot Branching in Clerodendrum japonicum
Source: Plants (Basel). 2026 Sep 15;15(18):2827. doi: 10.3390/plants15182827 (PMC13610545; doi:10.3390/plants15182827)
Supplement: Supplementary file 1 [file plants-15-02827-s001.zip › Figure S1.pdf]

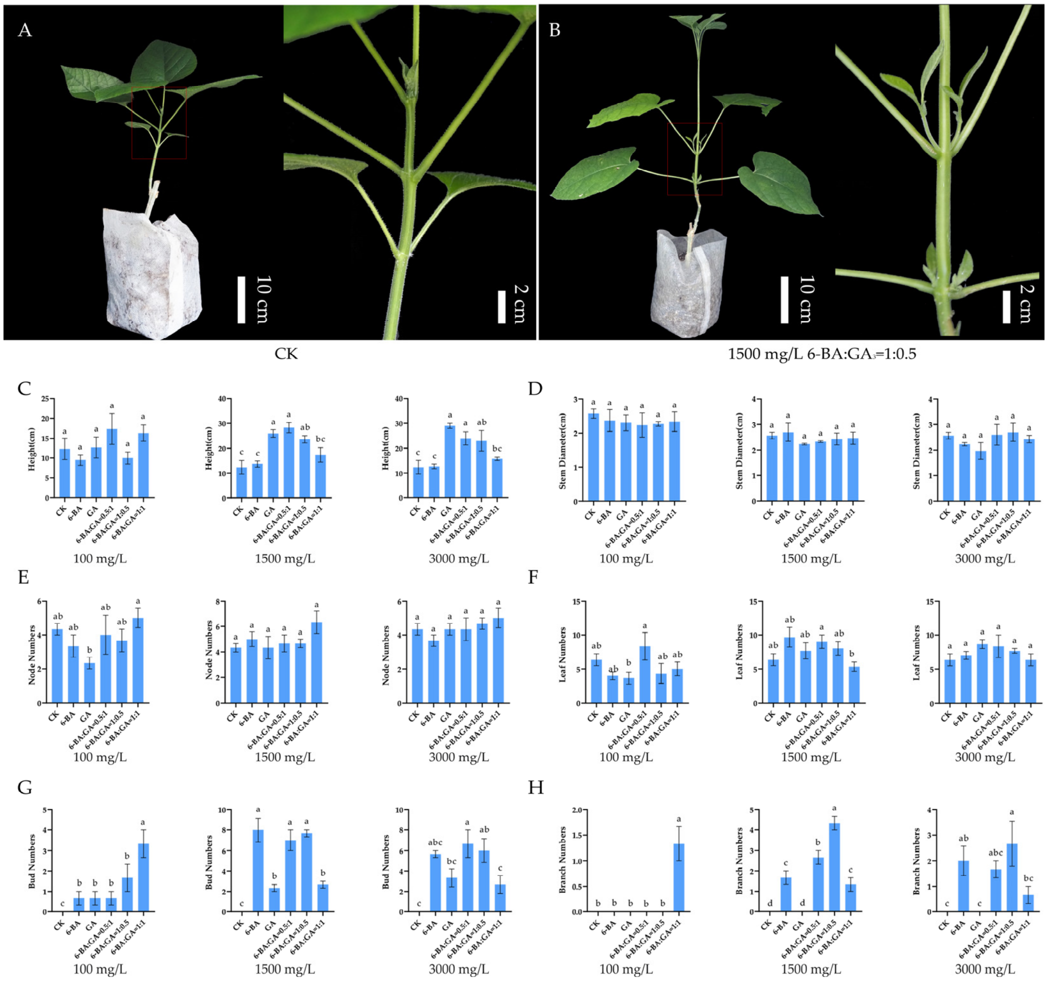

Supplementary Figure S1. Combined treatment regulation of branching traits by GA_3 and 6-BA in *Clerodendrum japonicum*. (A–B) Representative phenotypes of *C. japonicum* seedlings treated with CK and the optimal hormone combination (1500 mg L^{-1} 6-BA:GA_3 = 1:0.5). The enlarged images on the right show the axillary bud and stem node regions corresponding to the boxed areas in the whole-plant images. (C–H) Effects of different hormone concentrations and 6-BA:GA_3 ratios on plant height (C), stem diameter (D), node number (E), leaf number (F), bud number (G), and branch number (H). Seedlings were treated with three hormone concentration levels (100, 1500, and 3000 mg L^{-1}) under different 6-BA:GA_3 combinations. Data are presented as mean ± SD (n = 3). Different lowercase letters indicate significant differences among treatments according to one-way ANOVA followed by Duncan's multiple range test ($P < 0.05$).
