# Supplementary material for: Integration of Axillary Meristem Development and Gibberellin Homeostasis by CjLAS Regulates Shoot Branching in Clerodendrum japonicum
Source: Plants (Basel). 2026 Sep 15;15(18):2827. doi: 10.3390/plants15182827 (PMC13610545; doi:10.3390/plants15182827)
Supplement: Supplementary file 1 [file plants-15-02827-s001.zip › Figure S2.pdf]

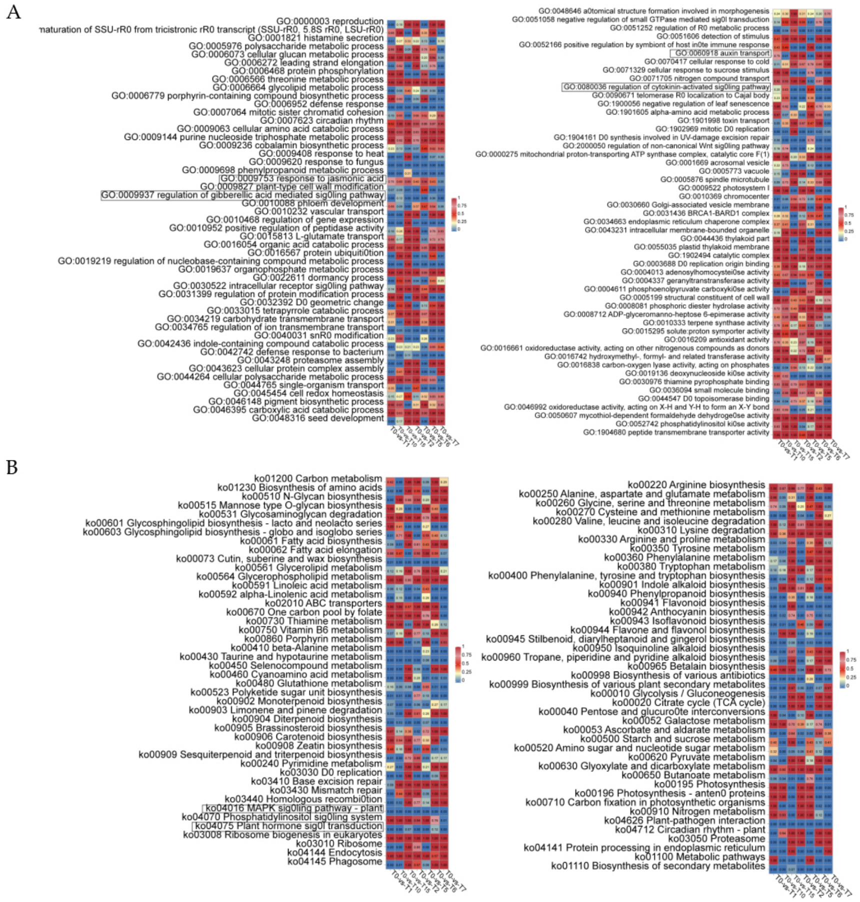

Supplementary Figure S2. Functional enrichment analysis of DEGs during hormone-induced branch development based on GO and KEGG pathways. (A) GO enrichment analysis of DEGs showing significantly enriched biological processes, cellular components, and molecular functions. (B) KEGG pathway enrichment analysis of DEGs involved in hormone-responsive and developmental processes. Heatmaps illustrate the enrichment patterns of significantly enriched terms and pathways among different hormone treatments. Color gradients indicate the relative enrichment level.
