# Supplementary material for: Integration of Axillary Meristem Development and Gibberellin Homeostasis by CjLAS Regulates Shoot Branching in Clerodendrum japonicum
Source: Plants (Basel). 2026 Sep 15;15(18):2827. doi: 10.3390/plants15182827 (PMC13610545; doi:10.3390/plants15182827)
Supplement: Supplementary file 1 [file plants-15-02827-s001.zip › Figure S3.pdf]

1	CGACCCAGCTTGCATGCCTGCAGGTCGACGATTCGTTATGGCTGGCTTCT
51	TGTTCAACTATGGTGACTATGTTTGGTGCTTGATCTCTAATCATAGTCAA
101	CAGGCTTCCGAGACAGCTCCCAGGGACTCGGTGGAGGCGGTTAACCGAGT
151	TAACAGCAAGAGCTTCTCCCACTCTTCTGTTATACATGTGGGGTTTCAGA
201	TCTTCAAGTTCTTCACCAACGGGATGGAATTCAAAAGGGACATGAAGAGA
251	GTGGGCCAGATCAGCTAAACACCTCCCTGTTTCTATGACAGACTCAAGAC
301	ATGGGCCCACCCCGGTTATTCGGAGAAACGGGGCACCACCTGGTCTTGCA
351	GCTAGGGCCTGCATGAAAGCAGGCCATTGGTAACCTTGGAGAATATCGAG
401	ATCGATGATGTGGACACGCTCCTCAGCCTCAAAGGCTTCGAAAATTGCTT
451	GATTGGCAGTGAAGTGAGCGAATTTGATGTAGGGGCAGGCCTGATATAGG
501	ATCTGGTAAATCTTGAGGATTTCTAGGTTGTTAGCAGGAAAGGCAGCAAG
551	AATGGCTGAATCGTCAGATTGATCGGCAGCTGCCACAGCCGCCGCCGAGA
601	ATGCAAAGACAACCACCACAAGTTCATCAGGAAAATCAGATGCAGAATAT
651	GCATAATACTTTGATGGTGCCTATTATTGAACCTGAGCAGGTGATCAATT
701	ACCTGATGAAAGTGTACCCATATGTCCATGAAAAAAGATACGAAGGGGAT
751	ATAGTCACATATACCCCAAAATTACAAAAAAGTCAACAATCTTTATCGAA
801	CAGATATGTGGGGTGAGTTAAACGGTGACCTTCAGAAGTTTGATACAGTG
851	GATTTCTCAAGCTTCACATGCTCGAGACAACCGCTATCTCAAGCTTCTGC
901	TTTGAGTTTGATTACGTAAGGTAGCAGTGTCCTTTCATGTACCATTTTGG
951	CTAATACTCATTTTTCTTCGAAATCTTGCTGAATTCCATTTATCAGAACC
1001	TTAT**ATG**

Supplementary Figure S3. Promoter sequence of *CjLAS* in *C. japonicum*. The 1007 bp upstream promoter region of *CjLAS* was cloned from *C. japonicum*, and the obtained nucleotide sequence is presented. The cloned promoter region was used for subsequent cis-element analysis to investigate potential regulatory mechanisms controlling *CjLAS* expression.
