# Supplementary material for: Integration of Axillary Meristem Development and Gibberellin Homeostasis by CjLAS Regulates Shoot Branching in Clerodendrum japonicum
Source: Plants (Basel). 2026 Sep 15;15(18):2827. doi: 10.3390/plants15182827 (PMC13610545; doi:10.3390/plants15182827)
Supplement: Supplementary file 1 [file plants-15-02827-s001.zip › Table S1.pdf]

Supplementary Table S1. Primerinformation.

Gene	forword/ReverseprimerSequence(5'-3')	Purpose
CjUBQ	F:GGACTTCCCGAGTTTGAACAGGTG<br>R:TGGATGGCAGGCGTATTCATTATGTC	RT-qPCR
CjLAS	F:GTCCCACTGAGTTCAAATGCAG<br>R:CCAACAGCAAACACCCCTTATC	RT-qPCR
CjSMXL6	F:AGTCCATCAAAGATGCAGTGG<br>R:TTCTTGCTGGAAGACACACC	RT-qPCR
CjBRC1	F:CAAAGAATCCCATGTTGCTCCTC<br>R:AGAACACACCATTTCCCTCCTG	RT-qPCR
CjMAX2	F:GTTCACGCAGGAGGATGCTA<br>R:AGTGCTGGACCACTCTCTCT	RT-qPCR
AtActin2	F:TCTCCCGCTATGTATGTCGC<br>R;GTCACGTCCAGCAAGGTCAA	RT-qPCR
ATGA20ox1	F:GTTTTGCGACGACATGAGCC<br>R:CACCTTCCTGCAACCACAAA	RT-qPCR
ATGA20ox2	F:AACCGGCTTGTTATACCGGA<br>R:CTGGTGTTTGGCATGGAGGA	RT-qPCR
ATGA3ox2	F:CGTTATTGGCTCTCCCCTCC<br>R:GTCAACTGTACTAACGTCCACA	RT-qPCR
ATGA2ox2	F:GGCTTTGCTTAGAGTTTGACC<br>R:ACACTAGTTTTAACACGTACAACGC	RT-qPCR
ATGA2ox6	F:ATCGTTGGTTCTTGGTTGCG<br>R:AGCCGTGTCTTCTGGTCATT	RT-qPCR
ATGID1a	F:ACTGATGTTTGCGATTATTGGGT<br>R:AGGCAAGGAGAAGCACAGAG	RT-qPCR
ATRGA1	F:AAGCTGTTGCGGTGAACTCT<br>R:GACCTTCACAAGCCACCAGA	RT-qPCR
CjLAS	F:GGGGACGAGCTCGGTACCCGGGGATCC<br>ATGGCTAAAATGTATATACA<br>R:TCCTCGCCCTTGCTCACCATGTCGACGC<br>ATCGCCAAGCAGAAGCA	Subcellular Localization
CjLAS	SP1:TGCTTGATTGGCAGTGAAATGAGC<br>SP2:GGAGGAAAAGGGTTGAATGGTCTTG<br>SP3:ACCCGGTTAAGGTGGTGGAGAT	Genome walking
*CjLAS*_CDS	F:TCTAGAATGGCTAAAATGTATATACAG<br>GAAC<br>R:AAGCTTGTCGACGCATCGCCAAGCAGA<br>AGCA	Plant overexpression
CjLAS	F:GGAGGCCAGTGAATTCATGGGATCCAT<br>GGCTAAAAT<br>R:CGAGCTCGATGGATCCGCATCGCCAAG<br>CAGAAGCAG	Yeast two hybrid

Gene	forword/ReverseprimerSequence(5'-3')	Purpose
CjSHR	F:GGAGGCCAGTGAATTCATGGATACTTTGTTCACACTTTTCA<br>R:CGAGCTCGATGGATCCGGTACCTCAGGGTTTCCATG	Yeast two hybrid
CjGA20ox1	F:GGAGGCCAGTGAATTCATGACCATTAACTGTATGATAACC<br>R:CGAGCTCGATGGATCCGGTACCTCACCCCAAGTGTT	Yeast two hybrid
CjLAS	F:AGGCCTGGCGCGCCACTAGTGGATCCATGGCTAAAATGTATATACAGG<br>R:TATGGGTACATCCCGGGAGCGGTACCGCATCGCCAAGCAGAAGCAG	Bimolecular fluorescence complementation
CjSHR	F:AGGATCTTGGGCCCAGGCCTACTAGTATGGATACTTTGTTCACACT<br>R:TCGAGCTCCTACCCGGGAGCGGTACCTCAGGGTTTCCATG	Bimolecular fluorescence complementation
CjGA20ox1	F:TTGGGCCCAGGCCTACTAGTGGATCCATGACCATTAACTGTATGAT<br>R:TCGAGCTCCTACCCGGGAGCGGTACCTCACCCCAAGTGTT	Bimolecular fluorescence complementation
