# Supplementary material for: Integration of Axillary Meristem Development and Gibberellin Homeostasis by CjLAS Regulates Shoot Branching in Clerodendrum japonicum
Source: Plants (Basel). 2026 Sep 15;15(18):2827. doi: 10.3390/plants15182827 (PMC13610545; doi:10.3390/plants15182827)
Supplement: Supplementary file 1 [file plants-15-02827-s001.zip › Table S2.pdf]

**Supplementary Table S2. Quality control analysis results.**

| Sample | Total Genes | Sequenced Total Genes | Raw Reads | Clean Reads | Q30(%) | GC Content(%) | Multiple_Mapped(%) | Total_Mapped(%) |
|---|---|---|---|---|---|---|---|---|
| all | 137206 | 136790 (99.70%) | | | | | | |
| T0-1 | 137206 | 116892 (85.19%) | 49639382 | 49556594 | 97.44% | 45.23% | 42336426 (85.43%) | 48032181 (96.92%) |
| T0-2 | 137206 | 116294 (84.76%) | 44897584 | 44820322 | 97.35% | 45.24% | 38280972 (85.41%) | 43442162 (96.93%) |
| T0-3 | 137206 | 116235 (84.72%) | 45103920 | 45029196 | 97.29% | 45.22% | 38430750 (85.35%) | 43656578 (96.95%) |
| T1-1 | 137206 | 113588 (82.79%) | 43293782 | 43221700 | 97.22% | 45.30% | 37226215 (86.13%) | 42112003 (97.43%) |
| T1-2 | 137206 | 113700 (82.87%) | 50516946 | 50433474 | 97.33% | 45.27% | 43381824 (86.02%) | 49144595 (97.44%) |
| T1-3 | 137206 | 112734 (82.16%) | 41174602 | 41103754 | 97.27% | 45.35% | 35301007 (85.88%) | 39964614 (97.23%) |
| T10-1 | 137206 | 110873 (80.81%) | 40588950 | 40520836 | 97.30% | 45.44% | 35078060 (86.57%) | 39580931 (97.68%) |
| T10-2 | 137206 | 109763 (80.00%) | 38483618 | 38419902 | 97.36% | 45.46% | 33228805 (86.49%) | 37463744 (97.51%) |
| T10-3 | 137206 | 112237 (81.80%) | 52206120 | 52121460 | 97.44% | 45.46% | 45127941 (86.58%) | 50876004 (97.61%) |
| T15-1 | 137206 | 117169 (85.40%) | 47776934 | 47687774 | 97.36% | 45.45% | 40853617 (85.67%) | 46398980 (97.30%) |
| T15-2 | 137206 | 116447 (84.87%) | 39416836 | 39342422 | 97.46% | 45.45% | 33622149 (85.46%) | 38172292 (97.03%) |
| T15-3 | 137206 | 117534 (85.66%) | 45191236 | 45111952 | 97.44 | 45.23 | 38594840 (85.55%) | 43808474 (97.11%) |
| T2-1 | 137206 | 110115 (80.26%) | 43084494 | 43013824 | 97.35 | 45.24 | 36007997 (83.71%) | 41739477 (97.04%) |
| T2-2 | 137206 | 110859 (80.80%) | 48265512 | 48188076 | 97.29 | 45.22 | 40122528 (83.26%) | 46743346 (97.00%) |
| T2-3 | 137206 | 109252 (79.63%) | 39602110 | 39521168 | 97.22 | 45.30 | 33151187 (83.88%) | 38252708 (96.79%) |
| T5-1 | 137206 | 109245 (79.62%) | 41215344 | 41145814 | 97.33 | 45.27 | 35947565 (87.37%) | 40067150 (97.38%) |
| T5-2 | 137206 | 109837 (80.05%) | 47374080 | 47297486 | 97.27 | 45.35 | 41326511 (87.38%) | 46196827 (97.67%) |
| T5-3 | 137206 | 108903 (79.37%) | 41846184 | 41778808 | 97.30 | 45.44 | 36487111 (87.33%) | 40822460 (97.71%) |
| T6-1 | 137206 | 113010 (82.37%) | 40050668 | 39980534 | 97.36 | 45.46 | 34500885 (86.29%) | 38892134 (97.28%) |
| T6-2 | 137206 | 112673 (82.12%) | 39940650 | 39868380 | 97.44 | 45.46 | 34382187 (86.24%) | 38763830 (97.23%) |
| T6-3 | 137206 | 112481 (81.98%) | 41815674 | 41742888 | 97.36 | 45.45 | 35923583 (86.06%) | 40706689 (97.52%) |
| T7-1 | 137206 | 115627 (84.27%) | 46297094 | 46210466 | 97.46 | 45.45 | 39011936 (84.42%) | 44774033 (96.89%) |

| Sample | Total Genes | Sequenced Total Genes | Raw Reads | Clean Reads | Q30(%) | GC Content(%) | Multiple_Mapped(%) | Total_Mapped(%) |
|---|---|---|---|---|---|---|---|---|
| T7-2 | 137206 | 115151 (83.93%) | 42960222 | 42884692 | 97.50 | 45.48 | 36439444 (84.97%) | 41607303 (97.02%) |
| T7-3 | 137206 | 115313 (84.04%) | 40582242 | 40513292 | 97.40 | 45.00 | 34597772 (85.40%) | 39325083 (97.07%) |
